# A novel investigation of pressure-induced semiconducting to metallic transition of lead free novel Ba_3_SbI_3_ perovskite with exceptional optoelectronic properties

**DOI:** 10.1039/d4ra00395k

**Published:** 2024-04-08

**Authors:** Md. Ferdous Rahman, Md. Naim Hasan Toki, Ahmad Irfan, Aijaz Rasool Chaudhry, Rajabur Rahaman, Md. Rasheduzzaman, Md. Zahid Hasan

**Affiliations:** a Advanced Energy Materials and Solar Cell Research Laboratory, Department of Electrical and Electronic Engineering, Begum Rokeya University Rangpur 5400 Bangladesh ferdousapee@gmail.com; b Department of Chemistry, College of Science, King Khalid University P. O. Box 9004 Abha 61413 Saudi Arabia; c Department of Physics, College of Science, University of Bisha P. O. Box 551 Bisha 61922 Saudi Arabia; d Department of Electrical and Electronic Engineering, International Islamic University Chittagong Kumira Chittagong 4318 Bangladesh

## Abstract

The structural, electronic, mechanical, and optical characteristics of barium-based halide perovskite Ba_3_SbI_3_ under the influence of pressures ranging from 0 to 10 GPa have been analyzed using first-principles calculations for the first time. The new perovskite Ba_3_SbI_3_ material was shown to be a direct band gap semiconductor at 0 GPa, but the band gap diminished when the applied pressure increased from 0 to 10 GPa. So the Ba_3_SbI_3_ material undergoes a transition from semiconductor to metallic due to high pressure at 10 GPa. The Ba_3_SbI_3_ material also exhibits an increase in optical absorption and conductivity with applied pressure due to the change in band gap, which is more suitable for solar absorbers, surgical instruments, and optoelectronic devices. The charge density maps confirm the presence of both ionic and covalent bonding characteristics. Exploration into the mechanical characteristics indicates that the Ba_3_SbI_3_ perovskite is mechanically stable. Additionally, the Ba_3_SbI_3_ compound becomes strongly anisotropic at high pressure. The insightful results of our simulations will all be helpful for the experimental structure of a new effective Ba_3_SbI_3_-based inorganic perovskite solar cell in the near future.

## Abstract 2

The structural, electronic, mechanical, and optical characteristics of barium-based halide perovskite Ba_3_SbI_3_ under the influence of pressures ranging from 0 to 10 GPa have been analyzed using first-principles calculations for the first time.

## Graphical abstract

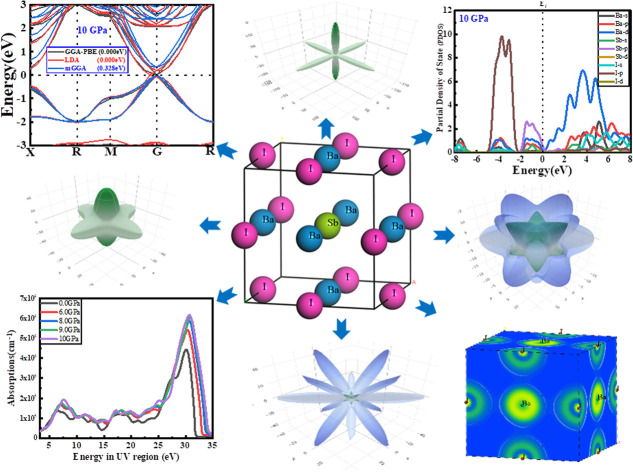

## 1. Introduction

In our contemporary world, notable for rapid population expansion and industrial development, the need for energy is progressively rising. To meet the demand and ensure a sustainable supply of energy, there is no alternative to finding a renewable source of energy. The pressing concern lies in the heightened demand for superior PV cells, optoelectronics, and electronic devices amid a growing global population, particularly in the face of exacerbated energy shortages.^1–4^ Metal-halide perovskites have exhibited significant potential in the application of photovoltaic (PV) cells. The remarkable optoelectronic properties, adjustable band gap features, absorption coefficient, conductivity, and mechanical stability of these materials have garnered considerable attention.^5,6^ The majority of perovskites show more promising characteristics than PV cells containing lead (Pb), and they tend to be environmentally-friendly.^7,8^ In a recent investigation, Ba_3_SbI_3_ showed outstanding optical, mechanical, and electronic properties for its usability in optoelectronic applications.^9^ The excellent photovoltaic efficiency demonstrated by the Mg_3_NF_3_ (*Pm*3*m*) prototype, resembling characteristics found in halide perovskites, closely aligns with its high-symmetry crystalline configuration, featuring a cubic lattice.^10^ This arrangement fosters a heightened likelihood of transitioning between p–s band edge states, contributing to the effective performance of photovoltaic systems.^11,12^ The pressure effect on perovskites is an interesting field for researchers nowadays.^13–16^ The pressure effect can tune various properties and cause a shift towards a metallic transition.^17,18^ Studying the characteristics of solids under pressure has recently gained popularity because it offers a thorough understanding of solid-state theories and an assessment of the values of key parameters. It is absolutely necessary to examine the physical properties of the Ba_3_SbI_3_ compound under high pressure because at high pressure different changes in physical phenomena may occur, such as phase transitions and changes in the physical and chemical characteristics of materials that happen at high pressure. Not only that, but investigation under hydrostatic pressure is usually required to evaluate the structural applications of materials. To the best of our knowledge, research on how high pressure affects the physical characteristics of Ba_3_SbI_3_ has not yet been conducted. The study of the various characteristics of compound Ba_3_SbI_3_ under high pressure is therefore very important.

Our main objective is to elucidate the response of Ba_3_SbI_3_ to applied pressure. Building on a prior study highlighting its novelty, our efforts are focused on discerning the amplified properties and exploring any potential metallic transitions in the material.^19^ In this study, we have applied 0 to 10 GPa (gigapascal) hydrostatic pressure. Higher pressure than 10 GPa can be applied, but we found a 0 eV band gap at 10 GPa, which is sufficient to suggest the metallic transition of the material.^18^ As Ba_3_SbI_3_ is non-toxic and provides a balanced photovoltaic performance, we have tried to enhance its optical, electronic, and mechanical properties to increase its performance by applying pressure from 0 to 10 GPa in this study. To examine the pressure effect on the material we used density functional theory (DFT).^20–22^ With CASTEP which uses DFT, by first principles calculation we have applied various pressures and optimized the cell to analyze its properties.^23–26^

## 2. Computational details of new perovskite Ba_3_SbI_3_ under different pressures

Density functional theory (DFT) was used to study the characteristics of Ba_3_SbI_3_ by implementing by the Cambridge Serial Total Energy Package (CASTEP) using the plane-wave pseudopotential (PWPP) total energy approach technique.^14,17,27,28^ Exchange–correlation was addressed using the Perdew–Burke–Ernzerhof (PBE) generalized gradient approximation (GGA). The GGA-PBE scheme is used because it predicts binding and dissociation energies. It is more accurate and universally applicable.^24^ For optimization of geometry we employed the Broyden–Fletcher–Goldfarb–Shanno (BFGS) minimization technique, facilitating rapid exploration of the structure with the lowest energy.^26^ The plane wave basis set was employed to compute various physical properties. The cut-off energy used was 500 eV. A *k*-point 6 × 6 × 6 grid was utilized, and the Monkhorst–Pack method was applied to sample specific points throughout the Brillouin zone.^29^ The maximum ionic Hellmann–Feynman force was less than 0.01 eV, and the maximum displacement was 5 × 10^−6^ eV per atom. Spin–orbit coupling was not included and the SCF tolerance was 2.0 × 10^−6^ eV per atom.^30^ All the parameters mentioned above were utilized in the computation of electronic, optical, and mechanical properties. A maximum strain amplitude of 0.003 and maximum displacement of 4.0 × 10^−4^ were applied for the calculation of elastic properties. To calculate phonon dispersion, we used finite displacement in one large supercell with a cutoff radius of 3.0. ELATE was used for the 2D and 3D representation of the Young's modulus, shear modulus, and Poisson ratio.^31^

## 3. Result and discussion

### 3.1. Structural details of new perovskite Ba_3_SbI_3_ under different pressures

**Fig. 1:**
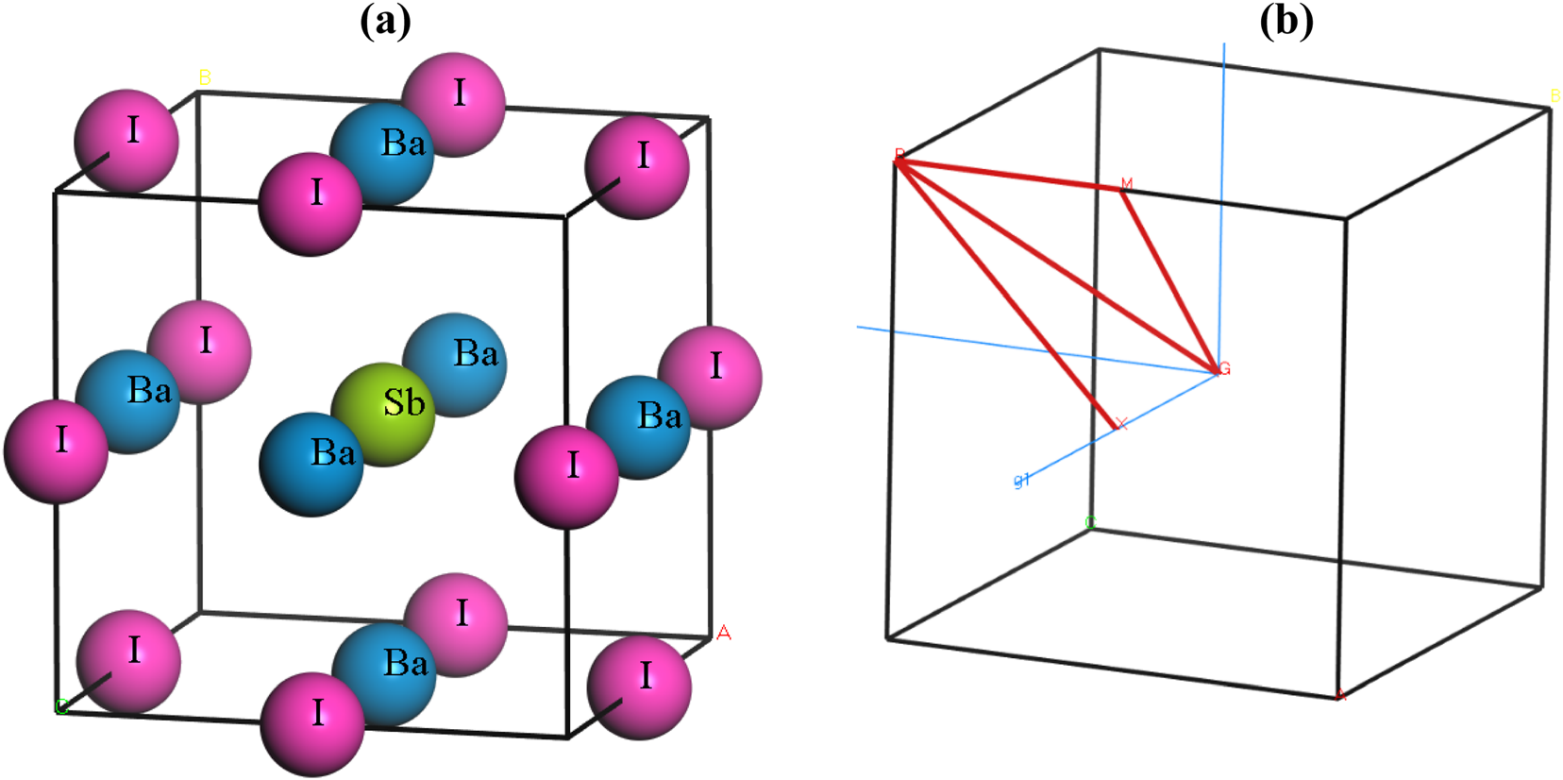
(a) The lattice structure of Ba_3_SbI_3_, and (b) the Brillouin zone path (*X*_*R*_*M*_*G*_*A*).

Ba_3_SbI_3_ is a cubic halide perovskite characterized by a high-symmetry structure within the space group *Pm*3*m* (#221).^32^Fig. 1(a) depicts the structure of Ba_3_SbI_3_. There are a total of 19 atoms in the unit cell. The Wycoff positions of the atoms are Ba (0.5, 0.5, 0) face centered, Sb (0.5, 0.5, 0.5) body centered, and I (0.5, 0, 0) edge centered. Fig. 1(b) shows the Brillouin zone path (*X*_*R*_*M*_*G*_*R*) which is represented by red lines. Table 1 presents the lattice constants, formation energy (final enthalpy), cell volume, and cell density. Table 2 presents the bond lengths of Ba–Sb, Ba–I, and Sb–I in Å.

**Table 1:** Lattice constant, volume, final enthalpy, and density with different pressure effects

Pressure (GPa)	Lattice constant (Å)	Volume (Å^3^)	Final enthalpy	Density (amu Å^−3^)	Ref.
0.0	7.05	350.403	−3199.64	2.609	9
0.0	7.04	350.091	−3199.65	2.612	This study
3.0	6.79	313.883	−3193.46	2.913	
6.0	6.62	289.819	−3187.82	3.155	
8.0	6.51	276.512	−3184.32	3.307	
9.0	6.48	271.606	−3182.61	3.367	
10.0	6.46	269.254	−3181.76	3.396	

**Table 2:** Bond lengths (Å) for Ba–Sb, Ba–I, and Sb–I

Pressure (GPa)	Ba–Sb (Å)	Ba–I (Å)	Sb–I (Å)	Ref.
0.0	3.525	3.525	4.985	9
0.0	3.524	3.524	4.894	This study
3.0	3.398	3.398	4.806	
6.0	3.309	3.309	4.679	
8.0	3.257	3.257	4.607	
9.0	3.238	3.238	4.579	
10.0	3.229	3.229	4.566	

From the summarized data in Table 1, it can be seen that the lattice constants and volume decrease with increasing pressure. On the other hand, as the volume is decreasing, the density is increasing along with the increase in pressure. Every formation energy shows negative values, which indicates thermodynamic stability at every applied pressure.^33,34^ The bond length decreases as the volume decreases, as summarized in Table 2, which suggests that the distance between the atoms is decreasing. This can lead to the band gap decreasing and an overlapping condition among the atoms.

### 3.2. Phonon dispersion of new perovskite Ba_3_SbI_3_ under different pressures

**Fig. 2:**
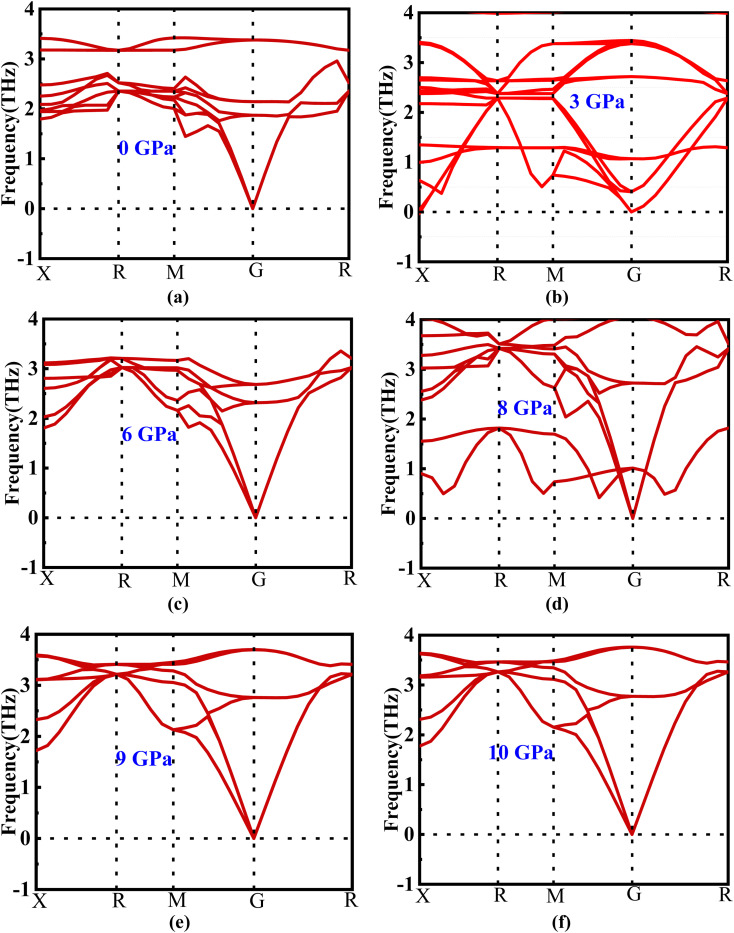
(a–f) Phonon dispersion of new perovskite Ba_3_SbI_3_ under different pressures.

The phonon dispersion curve of the new perovskite Ba_3_SbI_3_ under different pressures is shown in Fig. 2(a)–(f). From Fig. 2 we can observe that there is no negative frequency in the gamma point. Usually a negative frequency at the gamma point indicates the instability of the compound. As there is no negative frequency at the gamma point, we can say that Ba_3_SbI_3_ is a dynamically stable compound.^35^

### 3.3. Electronic properties

#### 3.3.1. Band structure of new perovskite Ba_3_SbI_3_ under different pressures

**Fig. 3:**
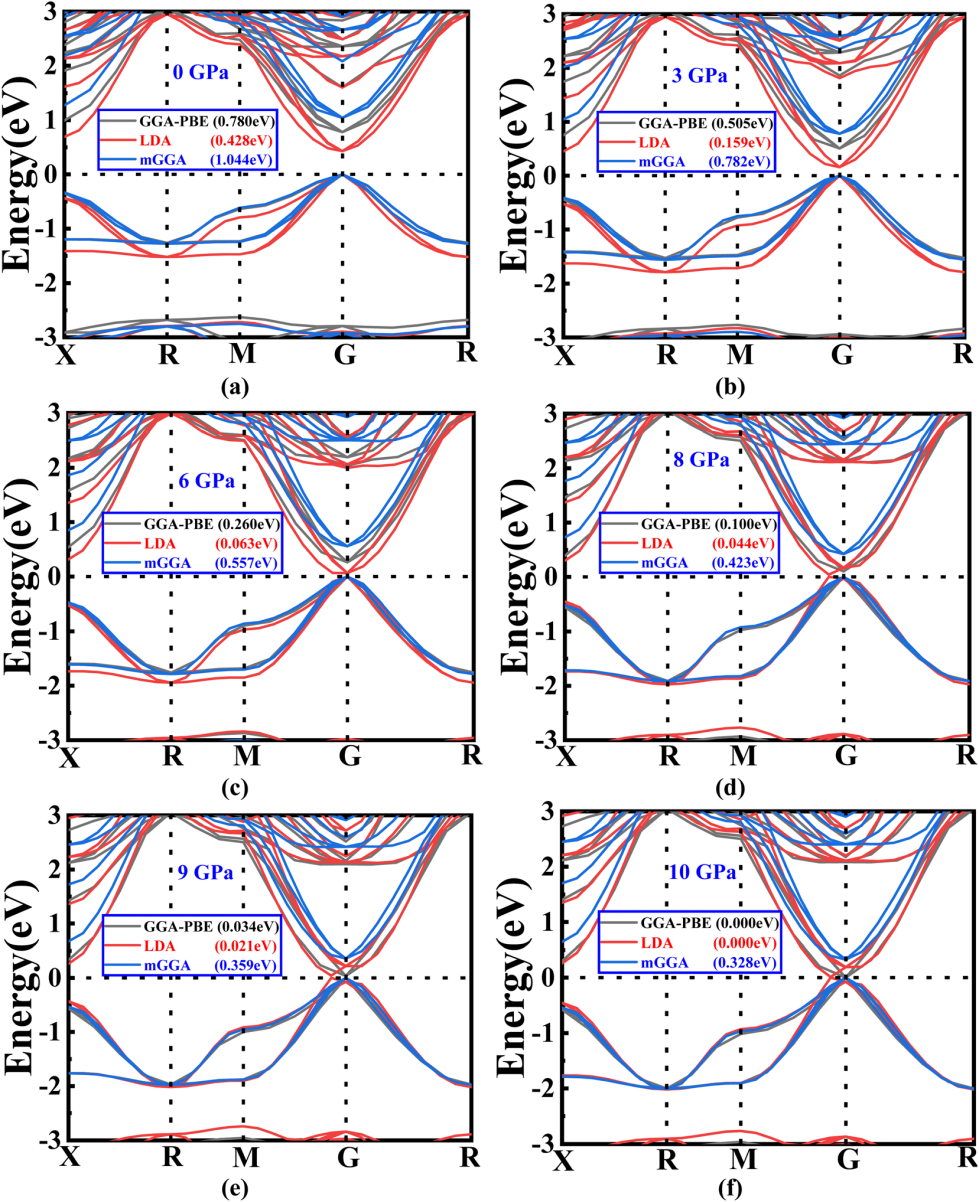
(a–f) The variations in band gap energy according to the change in pressure with different methods (GGA-PBE, LDA, and mGGA-RSCAN).

The electrical band structure of a material can be employed to attain a more profound insight into its essential physical traits, encompassing, but not limited to, optical properties and the behavior of charge transport in solids. It helps achieve significant understanding of its optical properties.^36^Fig. 3(a)–(e) represent the band structure of the Ba_3_SbI_3_ material at different pressures with Brillouin zone path *X*–*R*–*M*–*G*–*R*. We have shown the band energy range of Ba_3_SbI_3_ from −4 eV to 4 eV and the horizontal line at 0 eV is the Fermi level. All these data were generated using the GGA-PBE function.^37^ Nevertheless, Nayak and colleagues have recently reported that the collective trends in the alteration of the band gap (*E*_g_) and band structure under pressure remained unaffected by the choice of functional. They found that the PBE approach yielded fairly accurate results, implying its suitability for investigating material responses to pressure.^37,38^ Though the GGA-PBE function has some limitations, it underestimates the band gap but this is also found for the LDA + *U* and LDA techniques. To address the challenges in band gap calculations, we performed band structure calculations by employing three different schemes: GGA-PBE, LDA, and mGGA-RSCAN.^39^ Based on visual representations, it can be deduced that the LDA function yields a more underestimated band compared to GGA-PBE. Conversely, mGGA-RSCAN results in a higher band gap in contrast to GGA-PBE.

Notably, it is observed that the GGA-PBE and LDA functions exhibit a band gap of 0 eV at 10 GPa, distinguishing them from the mGGA-RSCAN functions in this specific pressure scenario. Ba_3_SbI_3_ has a direct band gap at *G* (the gamma point). The highest level of the valence band and the lowest level of the conduction band align at the *G* point. The direct band gap property ensures a higher absorption coefficient. In Fig. 3(a)–(e), we can observe that the band gap of Ba_3_SbI_3_ decreases with increasing pressure. At every pressure, it also shows a direct band gap structure. In Fig. 3(e) it is shown that there is no energy gap between the valence band and the conduction band. This leads the material to switch from a semiconducting nature to a conducting nature. Simply, we can say that increasing the pressure leads the compound to make a metallic transition, turning it into a good conductor. It increases the conductivity of the material. The narrow band gap of the Ba_3_SbI_3_ material not only facilitates the optimal absorption of visible light but also mitigates losses attributed to thermalization, wherein energy is dissipated as heat. Beyond this, its efficiency becomes particularly pronounced in the conversion of light into electricity. Moreover, this material boasts remarkable electron mobility, signifying the swift movement of electrons, and it excels at emitting light. These qualities collectively position it as a highly desirable material for integration into electronic and optical devices, showcasing its potential to enhance the performance and efficiency of such technological applications.^40,41^ With the band gap becoming narrower as the pressure increases, it can be asserted that the absorption of light will be maximized, especially at the peak pressure of 10 GPa.

**Fig. 4:**
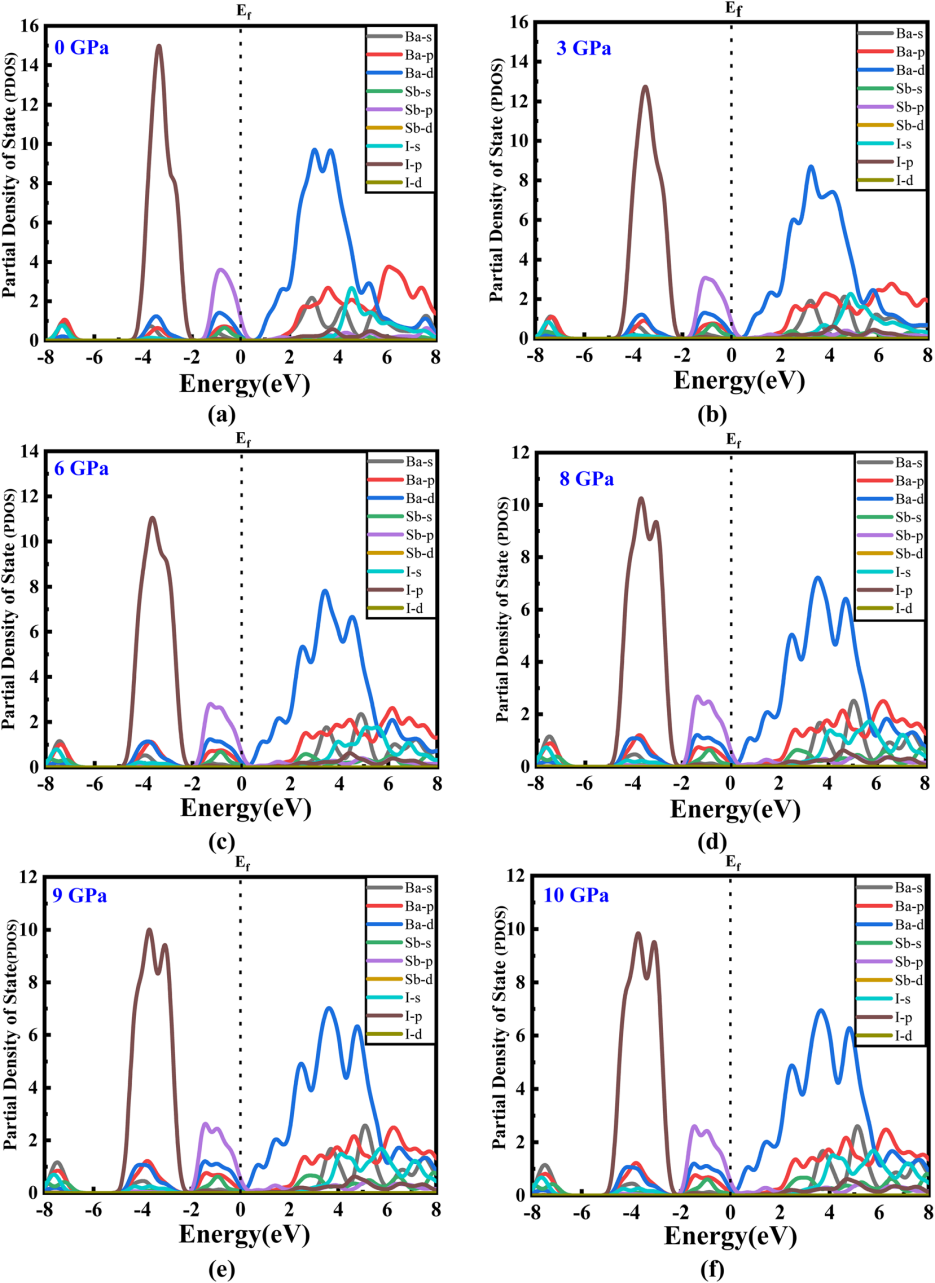
(a–f) The partial density of states of new Ba_3_SbI_3_ perovskite at different applied pressures.

The estimated partial density of states (PDOS) provides further insight into the electrical characteristics of Ba_3_SbI_3_. Fig. 4(a)–(e) represent the density of states with pressure. A vertical broken line indicates the Fermi level, *E*_F_ = 0 eV. From Fig. 4(a)–(e), the valence band for all the pressurized states is dominated by I-p and Sb-p. There is a slight contribution from Ba-d, Ba-p, and I-s. But, as the pressure increases, the contribution from I-p decreases noticeably. The impact of Sb-p has also dropped, but not as much as that of I-p.^42^ On the other hand, at the conduction band, Ba-d has the major impact. Ba-d makes a higher contribution than the others, such as Ba-p, Ba-s, or I-s. The share of Ba-d also reduces with increasing hydrostatic pressure.

**Fig. 5:**
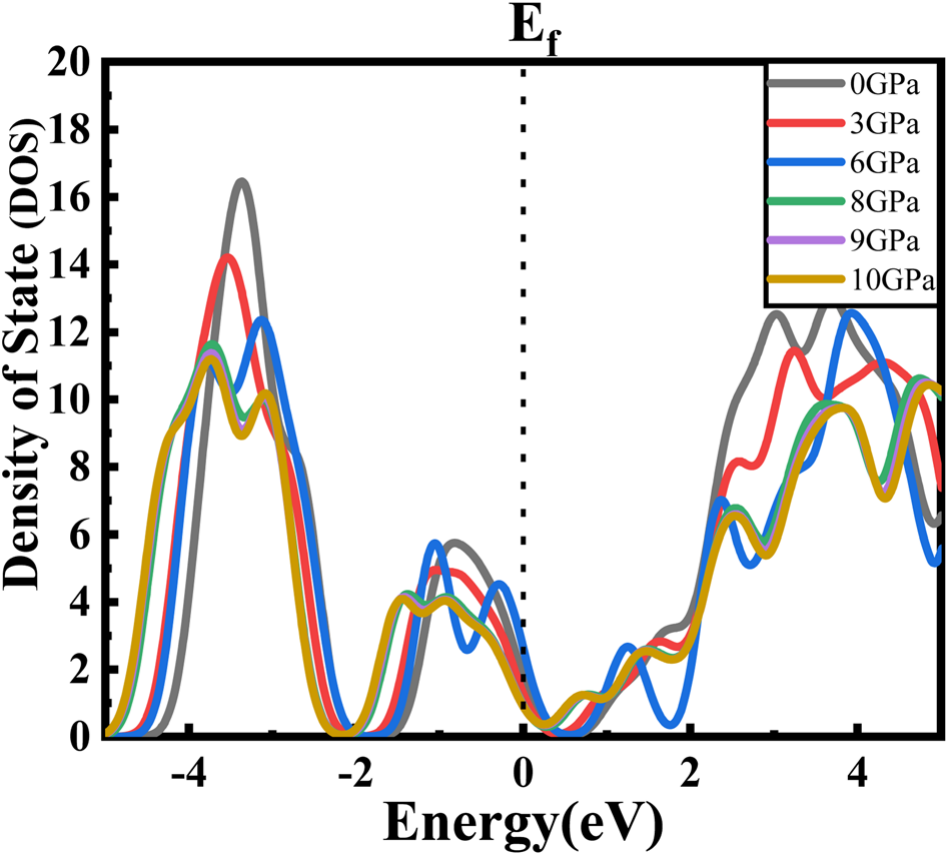
Total density of states of new Ba_3_SbI_3_ perovskite at different applied pressures.

For a clearer understanding, we have illustrated TDOS (the total density of states) in Fig. 5. As is clear, TDOS decreases with increasing pressure, and the above-mentioned orbital shows hybridization formation, which is depicted in the curve of the total density of states.^12,43–46^ We have finite values in every pressurized state, which indicates the metallic nature of the material.^34,41^ It can also be seen that the value at *E*_F_ decreases with pressure. We can say that the electronic state is lower at higher pressure, which means we may have less chance to have electrons at zero temperature at higher pressure than at ambient pressure.

#### 3.3.2. Charge density of new perovskite Ba_3_SbI_3_ under different pressures

**Fig. 6:**
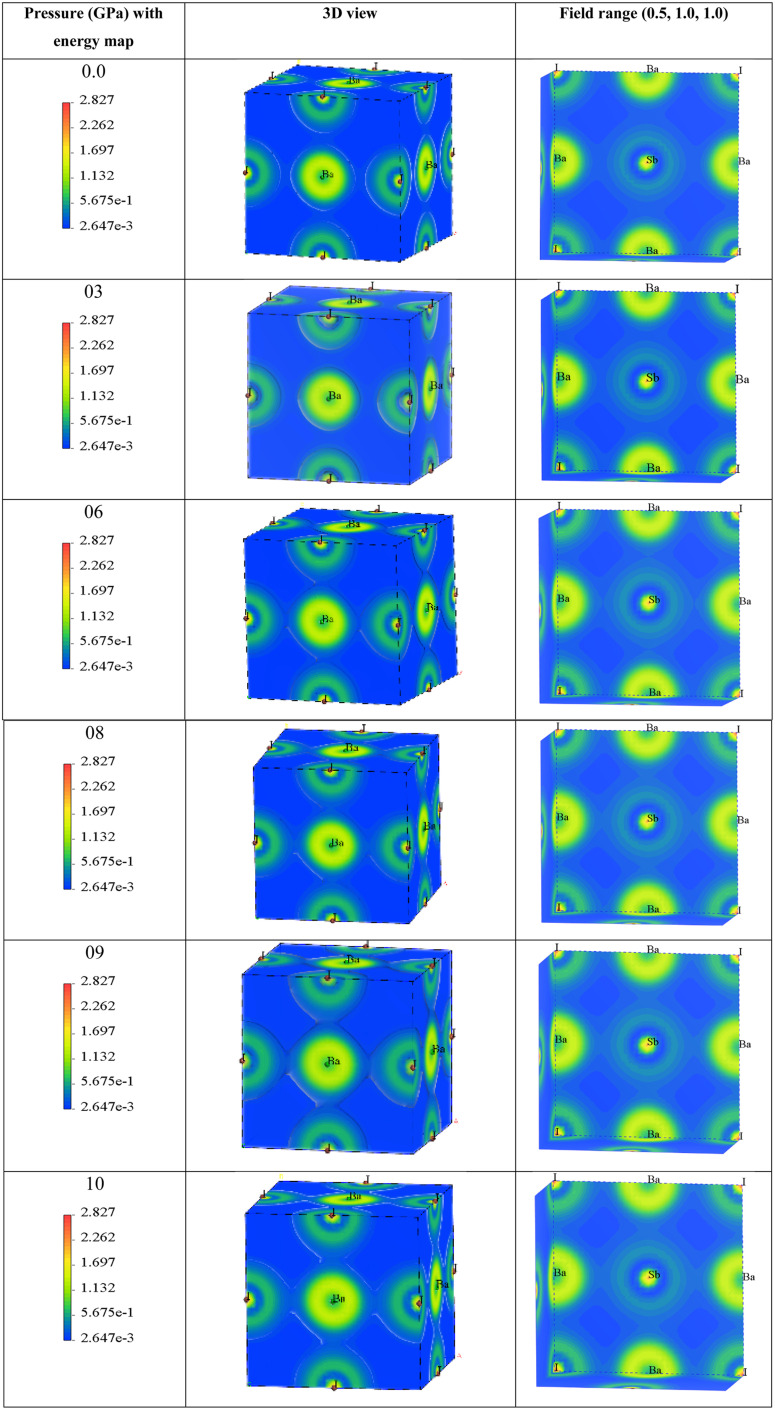
The variation in charge density according to different pressures, and energy mapping.

The distribution of charge density serves as a representation of the bonding nature among different atoms. By assessing the charge density, we aim to enhance our comprehension of the transfer of charge between atoms and the bonding characteristics within Ba_3_SbI_3_. For a better understanding of the nature of bonding between the atoms, we calculated the density of states.^9^Fig. 6 depicts the energy density of Ba_3_SbI_3_ in 3D view, with field range (0.5, 1.0, 1.0). Charge density is a detailed metric that reveals the complexities of the electric charge distribution across a specific surface area or encapsulated within a specific volume of a material or field. It reveals the nuances of charge concentration and arrangement, painting a vivid picture of how electric charge is intricately woven into the fabric of a specific domain.

The 3D representation shows the atomic arrangements of the lattice along with the charge distribution. It also represents the Ba–I bond. The field range (0.5, 1.0, 1.0) represents the nature of the Sb–I bond and the electron density field. Here, we can observe that at 0 GPa, the charge distribution between Ba–I and Sb–I atoms does not overlap, which confirms that the atoms have ionic bonds between them. Until a pressure of 6 GPa, there is no overlap seen in the depicted figures, but when the pressure increases to 6 GPa, a very small overlap can be seen in Ba–I, and it increases along with the increase in hydrostatic pressure. This means that there may also be bonding of a covalent nature. In the case of Sb–I, no overlap can be seen with the increase in pressure either. It keeps the ionic bonding nature between them, but all the electron charge density fields get closer to each other with the increase in pressure as the bond length is decreasing, which supports the results in Table 2. In Table 2, the bond lengths of Ba–Sb and Ba–I are less than that of Sb–I, so the fields between Ba–Sb and Ba–I seem to overlap but not significantly. Again, Sb–I retains its ionic nature after all the increases in hydrostatic pressure.

#### 3.3.3. Optical properties of new perovskite Ba_3_SbI_3_ under different pressures

In-depth studies of light absorbers, rectifiers, optical coatings, and comparable devices, designed to convert light into electrical energy, necessitate meticulous scrutiny of their intricate optical attributes. The examination encompasses a detailed exploration of the complex interplay between an externally applied electromagnetic wave and the material's capacity to respond to photons. This nuanced analysis not only reveals precise characteristics but also illuminates the material's potential applications intricately linked to its performance across the energy spectrum. A comprehensive investigation into these features entails a close examination of electronic transitions between occupied and vacant states, the complicated arrangements within band structures, the inherent nature of bonds, and the internal structural qualities of materials—all gleaned from their optical spectra.^47^

The Kramers–Kronig transformation is succeeded by the complex dielectric function, which exhibits dependency on either energy or frequency, expressed as eqn (1):

1 *ε*(*ω*) = *ε*_1_(*ω*) + *ε*_2_(*ω*)

Using *ε*_1_(*ω*) and *ε*_2_(*ω*), other optical properties such as the absorption coefficient *α*(*ω*), optical conductivity *σ*(*ω*), reflectivity *R*(*ω*), and refractive index *n*(*ω*) can be calculated using the following expressions (2)–(5):^48^2
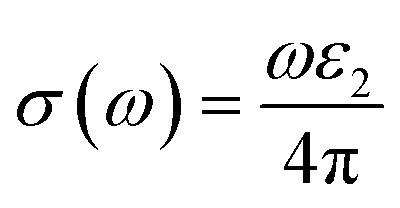
3
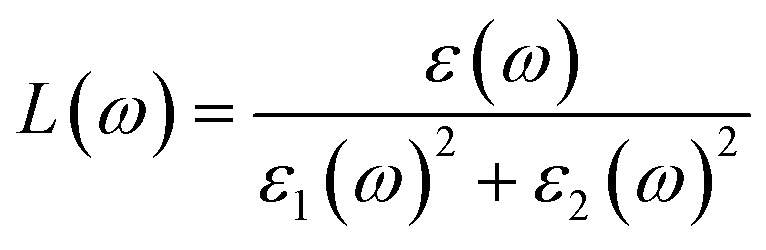
4
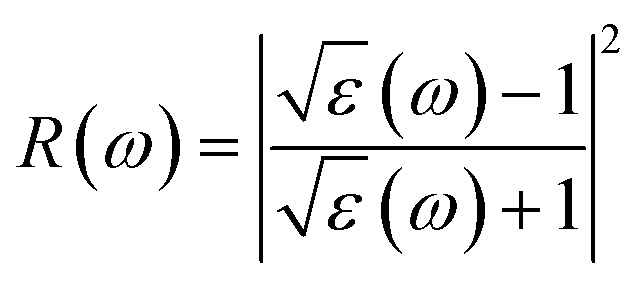
5
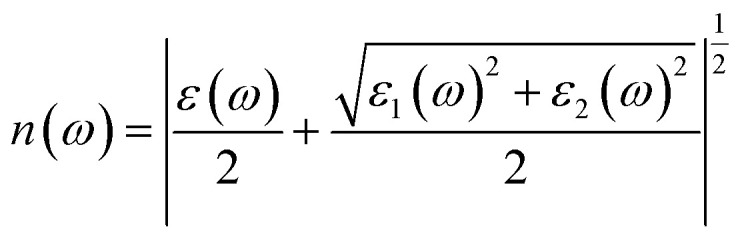


**Fig. 7:**
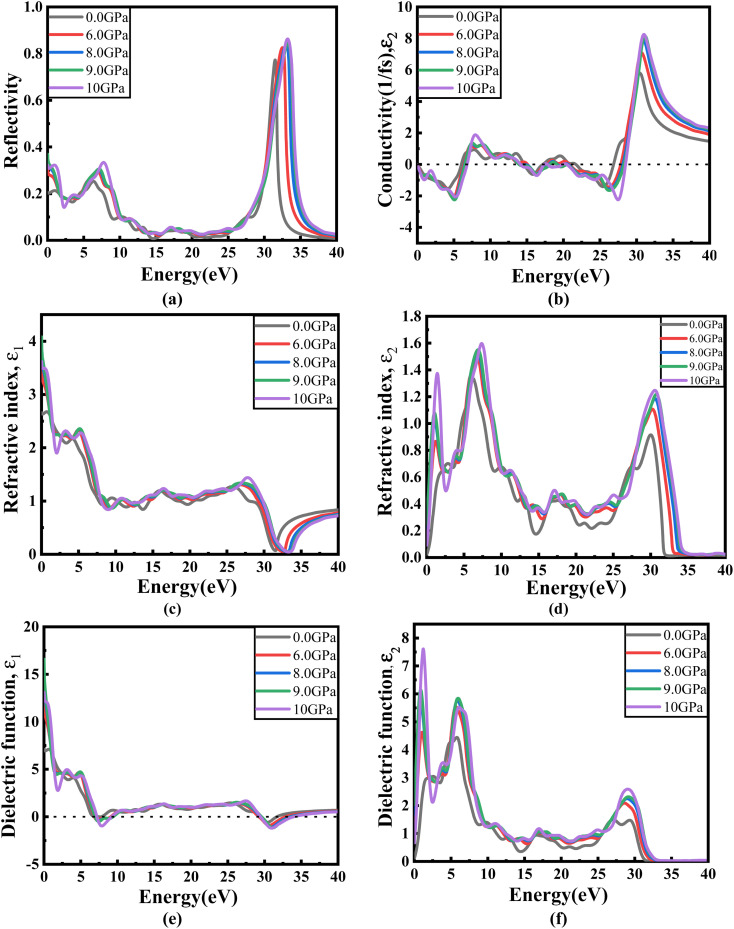
Energy-dependent (a) reflectivity, *R*(*ω*), (b) imaginary part *ε*_2_(*ω*) of conductivity *σ*(*ω*), (c) and (d) real and imaginary parts (*ε*_1_(*ω*) and *ε*_2_(*ω*)) of the refractive index, and (e) and (f) real and imaginary parts (*ε*_1_(*ω*) and *ε*_2_(*ω*)) of the dielectric function of the Ba_3_SbI_3_ compound under pressure.

**Fig. 8:**
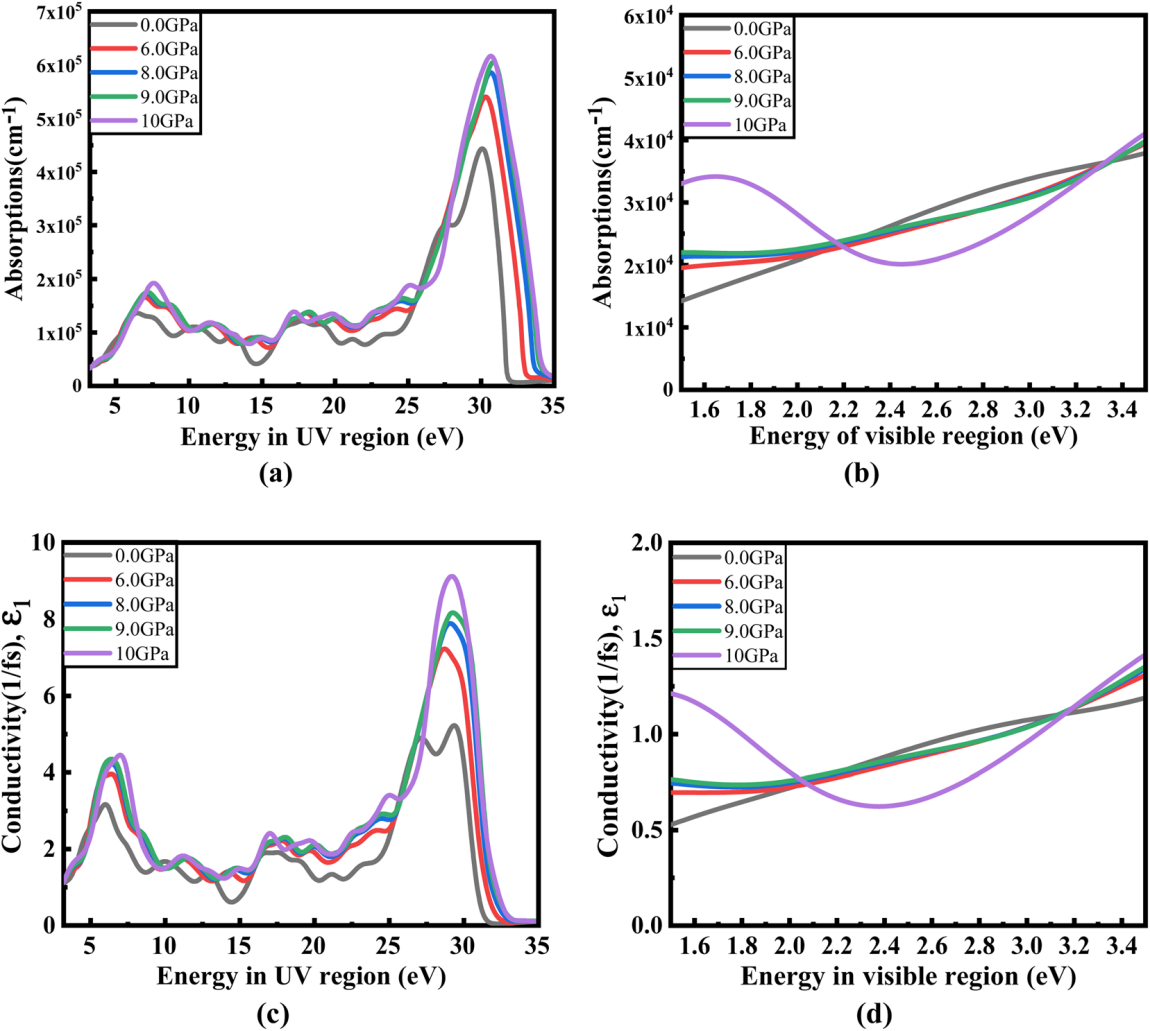
Change in (a and b) absorption and (c and d) conductivity according to the applied pressure in the visible and UV region.

Fig. 8(a) illustrates the optical absorption patterns of Ba_3_SbI_3_ perovskite. The optical absorption coefficient stands as a crucial parameter, offering insights into a material's capacity to absorb light energy. This information is pivotal for understanding the practical utility of the material, particularly in high-performance solar cells and other photovoltaic devices, as it directly correlates with the solar energy conversion efficiency of the material.^49,50^ The absorption index gets sharper as the pressure increases. Most of it is in the ultraviolet region, which is good for sterilizing surgical equipment.^51^ As the peak gets sharper, the material absorbs more rays in the UV region under higher pressure. This leads to its use in optoelectronic devices. The optical reflectivity in a material unfolds a rich narrative about its surface intricacies, presenting a detailed portrait of how effectively the surface can engage and reflect incident light. This analysis delves into the material's reflective potential, shedding light on the interplay between its surface nature and the incident light, thereby offering crucial insights into its optical characteristics.^52^ The heightened potential of metal halides for utilization in optoelectronic devices is indicated by the increased transparency (lower reflectance, *R*) within the visible energy range. Here, the reflectivity remains nearly the same for all pressures at lower energies, as shown in Fig. 7(a).

Optical conductivity serves as an alternative expression of photoconductivity, encapsulating the intricate behavior of materials when subjected to light. This phenomenon delves into the material's responsiveness to optical stimuli, offering a nuanced understanding of its conductive properties under the influence of light.^53^ The levels of both photoconductivity and electrical conductivity rise due to heightened photon absorption. The real part of the conductivity spectrum exhibits comparable features to the absorption spectrum, as illustrated in Fig. 8(c). This resemblance is attributed to the release of free carriers for conduction when the material absorbs energy. The optical conductivity increases under applied pressure, a consequence of the heightened absorption coefficient associated with increased pressure. The peaks in the real part of conductivity become more distinct under the influence of hydrostatic pressure, akin to the behavior observed in absorption. This is attributed to the rise in optical absorption within the investigated perovskite as pressure increases. The outcome is further corroborated by alterations in the band structure induced by pressure, where the band gap contracts with escalating pressure. On the other hand, the imaginary component becomes zero beyond a photon energy of 32 eV, as shown in Fig. 7(b).^53^ Here, the peak also gets sharper with pressure. This suggests that the material is demonstrating improved conductivity at elevated energies and pressures.

The refractive index (*η*) not only quantifies the shift in the speed of light within a material but also serves as a valuable gauge for anticipating the material's stability in applications involving various devices. This parameter encapsulates the intricate interplay of light within the material, offering crucial insights into its optical behavior and suitability for diverse technological applications.^54,55^Fig. 7(c) and (d) illustrate the changes in the real part and the imaginary part of the refractive index (*η*) induced by applied pressure. The extinction coefficient, an imaginary component of the refractive index, gauges the reduction in electromagnetic radiation within the material, while the real part establishes the phase velocity of electromagnetic waves within the material.^54^ As the pressure increases, both the real and imaginary parts of the reflectivity increase, which means that the material being under higher pressure reduces electromagnetic radiation and enhances the phase velocity of the electromagnetic wave.

The dielectric function is an essential factor associated with the rate at which charge carriers regenerate in specific materials utilized in solar cell applications.^18,56^ It offers a comprehensive insight into the operational effectiveness and capabilities of optoelectronic devices, serving as a crucial metric for assessing their performance in light-related applications. The dielectric function plays a pivotal role in optimizing and tailoring the design and functionality of these devices for improved efficiency and performance in diverse optoelectronic applications.^57^ The response of a material to incident light energy is referred to as the dielectric function. The peak of the static dielectric function stands out as a crucial parameter, furnishing essential information about the rate at which charge carriers recombine. This, in turn, offers a nuanced perspective on the overall efficacy and performance of optoelectronic devices.

By examining this specific aspect, one can gain a deeper understanding of how efficiently these devices operate in terms of managing and utilizing charge carriers, thereby influencing their overall potential in various applications within the realm of optoelectronics.^56,58^ Elevated dielectric constant values in perovskite solar cells may result in reduced rates of recombination. The real part of the dielectric function is depicted in Fig. 7(e). This component of the dielectric function exhibits an elevated value at lower photon energies and subsequently diminishes rapidly as the photon energy increases. It is commonly understood that a perovskite material featuring an elevated value in the real part of the dielectric function demonstrates a narrower band gap.^59^ Materials characterized by wide band gaps typically display a diminished static value of the dielectric constant.^60^ The peaks of the imaginary component of dielectric functions show a significant increase under pressure, providing validation for the absorption spectra results depicted in Fig. 7(f).

### 3.4. Mechanical properties of new perovskite Ba_3_SbI_3_ under different pressures

The elastic constants of solid materials play a pivotal role, serving as vital indicators that establish a substantial connection between mechanical properties. They offer valuable insights into the prevailing forces within solids, providing essential information about material stability and rigidity. This interrelation delves into the intricate characteristics of forces acting on the material, contributing significantly to our understanding of its mechanical behavior and structural integrity.^61,62^ These properties encompass aspects such as bulk and shear modulus, Young's modulus, ductility/brittleness, and anisotropic characteristics. In an exploration of a material's mechanical behavior, elastic constants (*C*_*ij*_) stand out as the most critical factors, offering a comprehensive understanding of its response to various forces and stresses.^62,63^ The theory of ‘finite strain’ is employed to ascertain the mechanical properties of materials.^64^Tables 1 and 2 show the lattice constants, volume, and bond lengths under increasing pressure. It is crucial to comprehend the impact of pressure on the values of *C*_*ij*_, as presented in Table 3.

**Table 3:** Elastic constants *C*_11_, *C*_12_, *C*_44_, Cauchy pressure *C*_p_, crystal stiffness *C*_s_, and Kleinman parameter *ζ*

Pressure (GPa)	*C*_11_	*C*_12_	*C*_44_	*C*_p_	*C*_s_	*ζ*	Ref.
0.0	53.817	7.0199	8.6732	−1.653	23.399	0.28	9
0.0	54.067	7.0366	8.6379	−1.601	23.515	0.28	This study
3.0	85.952	9.9245	8.3235	1.601	38.014	0.27	
6.0	115.642	11.6786	6.0778	5.592	51.981	0.25	
8.0	140.135	14.9781	6.2115	8.767	62.578	0.25	
9.0	142.133	12.3566	2.4194	9.937	64.888	0.24	
10	149.107	14.0308	3.3844	10.646	67.538	0.24	

The elastic constants *C*_*ij*_, represented as *C*_11_, *C*_12_, and *C*_44_, are computed for the Ba_3_SbI_3_ compound through deformation of the cubic unit cell using an appropriate strain tensor. These values are presented in Table 3. It is imperative for these constants to adhere to the conditions of equational inequalities, known as the Born stability criteria, in order to ensure mechanical stability:^65^

6 *C*_11_ − *C*_12_ > 0, *C*_11_ + 2*C*_12_ > 0 and *C*_44_ > 0

Again, the mechanical stability of a cubic cell is denoted by ‘*B*’,^66^ where:

7 *C*_12_ < *B* < *C*_11_

Applying mechanical stress to a material along a specific crystallographic direction, denoted *α*, involves utilizing *C*_11_ to quantify the material's resistance to stress. The elastic parameter *C*_12_, recognized as an off-diagonal shear component, characterizes the material's resistance to various forms of distortion. This parameter provides insights into the material's ability to withstand and respond to deformation. When a tangential stress is applied to the [1 0 0] plane along the [1 0 0] direction, the material's ability to resist shear deformation is expressed by the elastic constant *C*_44_.

Cauchy pressure:

8 *C*_p_ = *C*_12_ − *C*_44_

In the context of a compound, the Cauchy pressure represents a fundamental mechanical parameter essential for characterizing the material. Examining a material's Cauchy pressure value provides an additional method to discern whether the material exhibits brittle or ductile behavior.^67^

Crystal stiffness:9
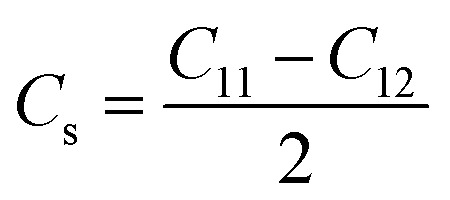


Crystal stiffness signifies the material's resistance to applied shear stress in the [1 1 0] plane along the [1 1 0] direction, indicating its ability to withstand shear deformation.

Kleinman parameter:10
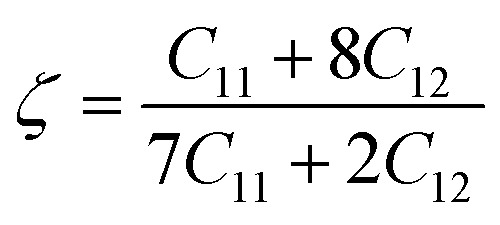


With a dimensionless range between 0 and 1, the Kleinman parameter signifies the extent of bond bending contributions. A lower value, closer to 0, suggests minimal bond bending, while a higher value, closer to 1, indicates the stretching of bonds in response to externally applied stress.^68^

From Table 3, it is clear that all the elastic constants increase with increasing pressure. The Cauchy pressure also increases. It shifts from negative to positive, which confirms that the material alters its nature from brittle to ductile. The ductility of the material increases with the increase in hydrostatic pressure. The crystal stiffness also increases, which means the material shows more resistance to shear deformation during the application of shear stress. Again, as the Kleinman parameter decreases with pressure, the bond bending becomes lower when pressure is applied.

Utilizing the Voigt–Reuss–Hill (VRH) technique, estimates have been made for the bulk modulus (*B*_H_), shear modulus (*G*_H_), Young's modulus (*Y*), and Poisson ratio (*ν*) of Ba_3_SbI_3_. This approach provides comprehensive insights into the material's mechanical properties, encompassing its responses to compression, shear, elasticity, and deformation characteristics. The derived values offer a detailed understanding of how Ba_3_SbI_3_ behaves under different mechanical forces.^69,70^

Bulk modulus:11
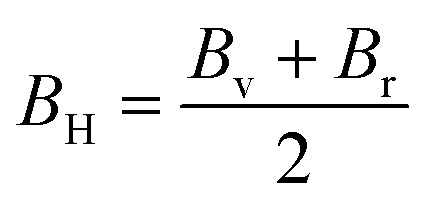


The measure of a material's ability to withstand compression is determined by its bulk modulus. In the elastic range of a material, it represents the relationship between compressive pressure and volumetric strain. Bulk modulus is denoted by the symbol *B*, and its units are typically expressed in pascals (Pa) or gigapascals (GPa).^71^

Young's modulus:

Young's modulus, or the modulus of elasticity, serves to quantify a material's ability to resist tension or compression along its length. The expression for Young's modulus is^72^12
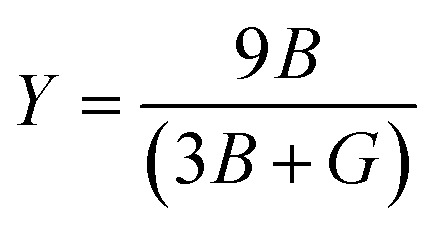


Shear modulus:13
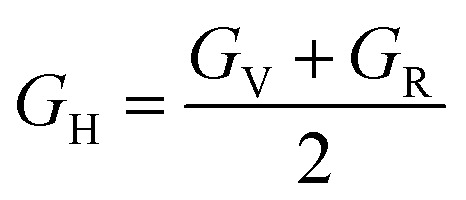


Also referred to as the modulus of rigidity, the shear modulus gauges a material's capacity to withstand shear stress.^73^

From Table 4, we get an idea of the Young's, shear, and bulk moduli (Voigt–Reuss–Hill). It is clear that increasing pressure increases *Y*_H_, *G*_H_, and *B*, so it is more likely that under hydrostatic pressure, Ba_3_SbI_3_ will have greater ability to resist tensile compression of its length, fracturing, and plastic deformation. The fact that *Y*_V_, *Y*_R_, and *Y*_H_ are not nearly equal to each other suggests that Ba_3_SbI_3_ shows anisotropic behavior.

**Table 4:** Young's modulus (Voigt–Reuss–Hill), shear modulus (Voigt–Reuss–Hill), and bulk modulus

Pressure (GPa)	*Y* (GPa)			*G* (GPa)			*B* (GPa)	Ref.
	*Y*_V_	*Y*_R_	*Y*_H_	*G*_V_	*G*_R_	*G*_H_		
0.0	35.97	29.7	32.89	14.56	11.59	13.08	22.62	9
0.0	36.05	29.66	32.91	14.59	11.56	13.07	22.71	This study
3.0	50.88	32.59	42.03	20.19	12.10	16.15	35.26	
6.0	62.35	26.41	45.25	24.44	9.39	16.92	46.33	
8.0	73.79	27.56	51.84	28.76	9.71	19.23	56.69	
9.0	70.62	11.53	42.98	27.41	3.93	15.67	55.62	
10	74.87	15.89	47.16	29.05	5.46	17.25	59.06	

Pugh's ratio (*B*/*G*):

A direct relationship between shear and bulk moduli, represented by *B*/*G*, constitutes the definition of Pugh's ratio. This ratio functions as an indicator of a compound's brittleness or ductility. The pivotal threshold for Pugh's ratio is set at 1.75. Materials with a higher ratio, surpassing 1.75, are inclined towards ductility, whereas those with a lower value tend to exhibit brittleness.^74,75^

Machinability index:14
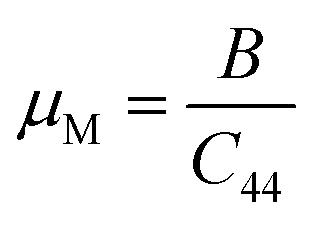


Machinability stands as a crucial parameter that assesses a material's aptitude for shaping with cutting tools. The machinability index assumes a pivotal role in establishing the ideal conditions for machine usage, cutting forces, temperature, and power strain to ensure the economically efficient machining of a material.^76^

Hardness factor:

15 *H* = 0.92*K*^1.137^*G*^0.708^

Comprehending the elastic and plastic traits of a material necessitates an understanding of its hardness.^77^

Poisson ratio:16
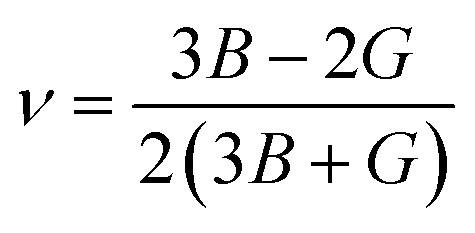


The Poisson ratio (*ν*) serves as a crucial parameter in assessing the mechanical attributes of crystalline solids. This value allows for the prediction of the material's stability in response to shear forces, with a lower *ν* suggesting enhanced stability against shear. The Poisson ratio significantly influences the interatomic forces within solids, playing a pivotal role in shaping their overall mechanical behavior. Solids within the range of 0.25 to 0.50 are indicative of being dominated by central forces. Beyond this range, it characterizes solids where central forces are not dominant.^77^ The Poisson ratio serves as a crucial tool for anticipating the failure mode of crystalline solids. Materials with a Poisson ratio below the critical threshold of 0.26 are anticipated to exhibit brittleness, while those surpassing this threshold are predicted to display ductile characteristics.^78^

Table 5 shows some of these other mechanical parameters, like Pugh's ratio, machinability index, hardness factor, and Poisson ratio. It is clear from the Pugh's ratio that the value increases with increasing pressure. From 3.0 GPa to 10 GPa, it is greater than 1.75, so it is clear that Ba_3_SbI_3_ shifts to ductility. This also supports the Cauchy-pressure transition. The value of *ν* is also greater than 0.26 from 3.0 to 10 GPa, increasing with applied pressure. This also supports the material's shifting nature from brittleness to ductility. Ba_3_SbI_3_ has a hardness of 2.42 at 0 GPa, which decreases with pressure, and at 10 GPa it is 0.2. The decreasing hardness factor suggests an improvement in the *μ*_M_ of Ba_3_SbI_3_. As the parameter *μ*_M_ increases with increasing pressure, this indicates that Ba_3_SbI_3_ exhibits superior lubricating properties, reduced feed friction, and heightened plastic strain.

**Table 5:** Pugh's ratio (*B*/*G*_H_), machinability index *μ*_M_, hardness factor *H*, and Poisson ratio *ν*

Pressure (GPa)	*B*/*G*_H_	*μ*_M_	*H*	*ν*	Ref.
0.0	1.729	2.6	2.44	0.235	9
0.0	1.737	2.63	2.42	0.258	This study
3.0	2.18	4.24	1.59	0.346	
6.0	2.738	7.62	0.73	0.337	
8.0	2.948	9.13	0.62	0.347	
9.0	3.549	22.98	0.12	0.371	
10	3.424	17.45	0.20	0.367	

### 3.5. Elastic anisotropy of new perovskite Ba_3_SbI_3_ under different pressures

The characteristics of a system may vary with direction, and the anisotropic index is employed for their calculation. For instance, the shear anisotropy factor is utilized to ascertain the level of anisotropy in the atomic bonding strength along different planes. The determination of three shear anisotropy factors (*A*_1_, *A*_2_, and *A*_3_) in a cubic system along the [100], [010], and [001] planes can be achieved through the following equation:^79^17
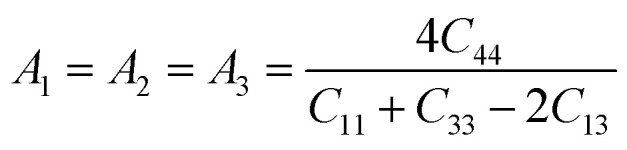


This is analogous to the Zener anisotropy factor (*A*), which is given by:^80^18
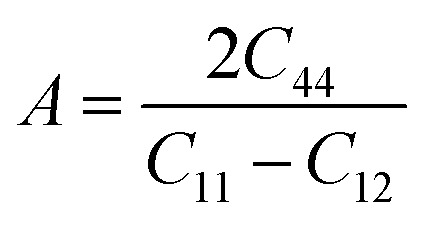


The concept of a universal anisotropy index, denoted *A*^U^, was initially introduced by Ranganathan and Ostoja-Starzewski:^81^19
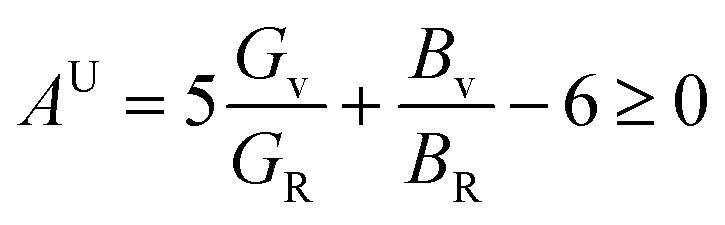


For an isotropic material, *A* = 1. The anisotropy refers to the variance from unity.^82^ The universal anisotropy index yields either zero or positive values, where positive values indicate the material's anisotropic characteristics, and a value of 0 signifies its isotropic properties.^83^ A solitary anisotropy index, in contrast to the multiple anisotropy factors assigned to various crystal planes, holds greater appeal due to its simplicity. It can be shown that the Ba_3_SbI_3_ compound exhibit elastic anisotropy, and there is a substantial increase in anisotropy with rising pressure.

Observing Table 6, we find that *A* is not unity, which refers to the anisotropic nature of Ba_3_SbI_3_. On the other hand, *A*^U^ is not 0 at any pressure. It increases with the applied hydrostatic pressure, which suggests the material undergoes massive anisotropic growth. The maximum and minimum ranges of Young's modulus, shear modulus, and Poisson ratio increased with pressure. The anisotropy index of these moduli also increased. For a better understanding, we have represented *Y*, *G*, and *ν* in 3D.

**Table 6:** Maximum and minimum value of Young's modulus (*Y*_max_ & *Y*_min_), Poisson ratio (*V*_max_ & *V*_min_), and shear modulus (*G*_max_ & *G*_min_); anisotropy value of Young's modulus, Poisson ratio, and shear modulus (*A*_Y_, *A*_V_, and *A*_G_), Zener anisotropy factor *A*, and universal anisotropy index *A*^U^

Pressure (GPa)	*Y*		*A*_Y_	*V*		*A*_V_	*G*		*A*_G_	*A*	*A* ^U^	Ref.
	*Y*_min_	*Y*_max_		*V*_min_	*V*_max_		*G*_min_	*G*_max_				
0.0	23.07	52.197	2.262	0.0593	0.546	9.2058	8.6730	23.399	2.698		1.28	9
0.0	22.99	52.446	2.280	0.0587	0.54863	9.3391	8.6379	23.515	2.722	0.036	1.31	This study
3.0	23.15	83.898	3.624	0.0348	0.69796	20.0138	8.3235	38.014	4.567	0.029	3.34	
6.0	17.47	113.5	6.497	0.0179	0.8227	45.9465	6.0778	51.982	8.553	o.119	8.01	
8.0	17.98	137.24	7.634	0.0162	0.84881	52.5260	6.2115	62.579	10.07	0.099	9.81	
9.0	7.156	140.16	19.59	0.0054	0.93843	175.319	2.4194	64.888	26.82	0.037	29.83	
10	9.963	146.69	14.72	0.0076	0.91906	120.675	3.3844	67.538	19.96	0.051	21.61	

**Fig. 9:**
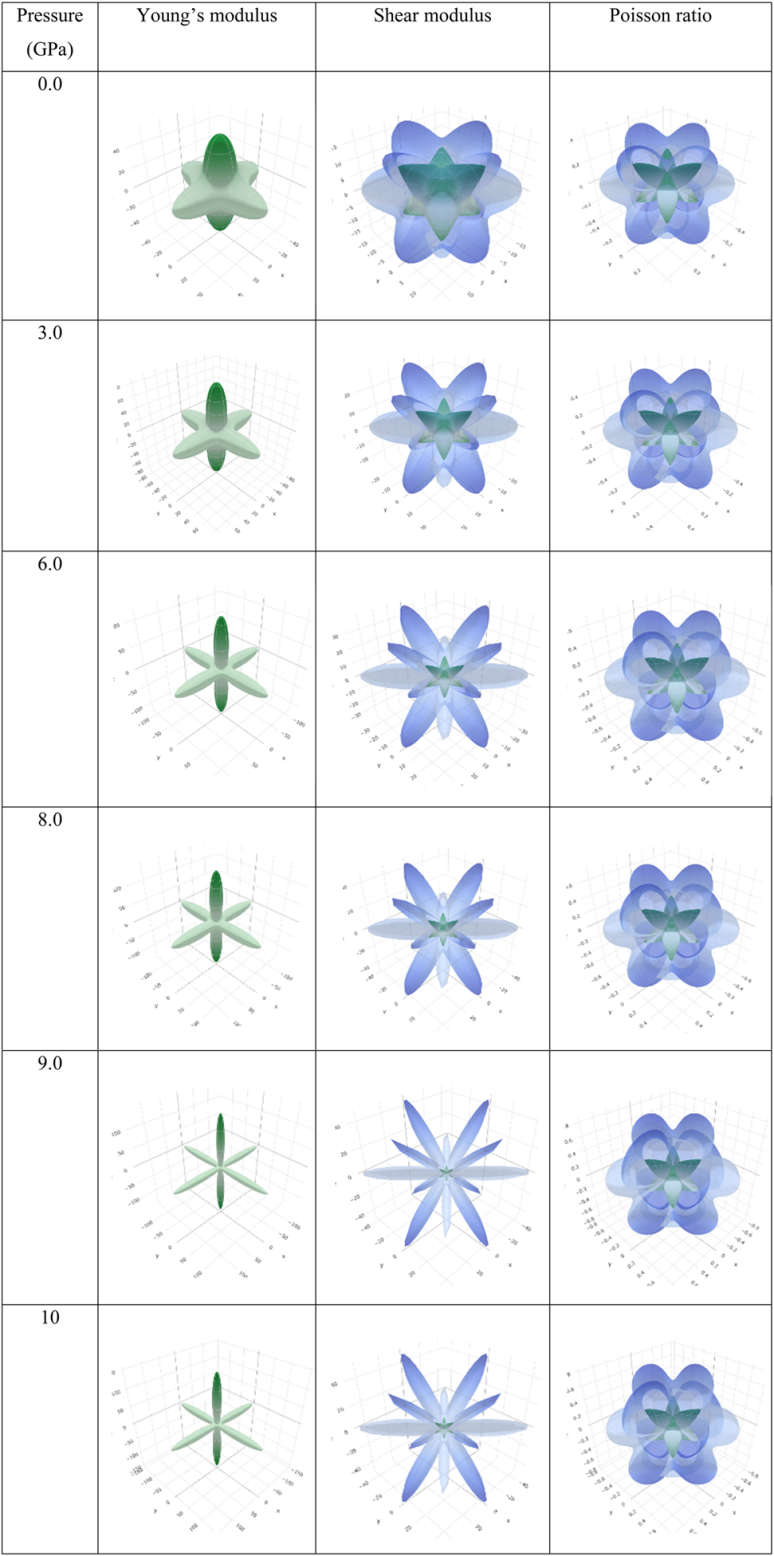
3D illustrations of Young's modulus, shear modulus, and Poisson ratio with different pressures of the new perovskite Ba_3_SbI_3_.

To depict the elastic anisotropy of the material, we utilized the ELATE method to ascertain the direction-dependent fluctuations of Young's modulus, shear modulus, and Poisson ratio, as shown in Fig. 9. This methodology contributes to a thorough comprehension of how the material's mechanical properties alter based on the direction of applied stress.

By acquiring insights into the directional elasticity of the material, we enhance our understanding of its responses to diverse mechanical stresses and its potential applications in various contexts.^31^ The three-dimensional spherical plots symbolize the isotropic nature, while any deviation from a spherical shape indicates the anisotropy of the material.^84^ The plots become sharper with increasing pressure. This shows that pressure enhances the anisotropic nature of Ba_3_SbI_3_.

## 4. Conclusions

The structural, electronic, optical mechanical, and elastic anisotropic properties of the Ba_3_SbI_3_ compound have been studied by applying pressures ranging from 0 GPa to 10 GPa with the help of the DFT method. At zero pressure, the calculated structural parameters of Ba_3_SbI_3_ show agreement with the available experimental and theoretical data. As the pressure increases, the lattice constants and volumes decrease. The band gap of Ba_3_SbI_3_ decreased to 0 eV at a pressure of 10 GPa, suggesting a metallic transition. The optical properties are also enhanced under pressure. It shows better absorption, conductivity, and dielectric constant under pressure, which mean the material can be used in optoelectronic devices, QLEDs, solar absorbers, and surgical instruments. Under the influence of pressure, the crystal elastic constant, *C*_*ij*_, fulfils the Born stability criteria which makes this compound mechanically stable. The Ba_3_SbI_3_ material is thermodynamically stable according to calculation of the formation enthalpy. From the calculated anisotropic indices, it is clear that the Ba_3_SbI_3_ compound becomes extremely anisotropic at high pressure. The perceptive results of all of these simulations will be useful for the experimental structure of an efficient new inorganic perovskite solar cell based on Ba_3_SbI_3_ in the near future.

## Ethical statement

All the authors declare that the manuscript does not have studies on human subjects, human data or tissue, or animals.

## Author contributions

Md. Ferdous Rahman: conceptualization, methodology, software, validation, formal analysis, visualization, investigation, data curation, supervision, writing-original draft, review & editing. Md. Naim Hasan Toki: methodology, software, validation, formal analysis, visualization investigation, data curation, writing-original draft, review & editing, Ahmad Irfan, Aijaz Rasool Chaudhry, Rajabur Rahaman, Md. Rasheduzzaman, Md. Zahid Hasan: validation, formal analysis, writing-original draft, review & editing.

## Conflicts of interest

The authors have no conflicts of interest.

## Supplementary Material
